# Editorial: Neurodegenerative diseases: From gut-brain axis to brain microbiome

**DOI:** 10.3389/fnagi.2022.1052805

**Published:** 2022-10-12

**Authors:** George Tetz

**Affiliations:** Department of Systems Biology, Human Microbiology Institute, New York, NY, United States

**Keywords:** brain microbiome, misfolding, Tau, beta-amyloid, TezR, gut-brain axis

This Research Topic was designed to explore the role of microbiota in neurodegenerative diseases. This topic is critical since it aims to shed light on the very first stages of neurodegenerative diseases. Understanding the multifaceted roles of microbiota in the development of these diseases enables the discovery of novel therapeutic targets. There are two major reasons why so many drugs for the treatment of neurodegenerative diseases have failed in the last few years (Yiannopoulou et al., 2019; Imbimbo and Watling, 2021). First, despite decades of research, the exact cause and trigger factors of these diseases have not been discovered, and neurodegenerative conditions are diagnosed when they are significantly advanced, yet the critical and irreversible pathogenic steps begins decades prior to the first clinical manifestations (Imbimbo and Watling, 2021; Sirkis et al., 2022). Second, it is impossible to develop a relevant animal model without knowledge of the exact pathogenesis. And without a clinically relevant animal model that can recapture not only familial, but also sporadic form of neurodegenerative diseases you cannot succed in clinical trials (Bjorkli et al., 2020). Therefore, understanding the triggering factors and protein misfolding in neurodegenerative diseases is the key to achieving a breakthrough in the successful prevention and treatment of neurodegenerative diseases.

Recently, microbiome-related triggering factors, such as bacterial extracellular nucleic acids and deoxyribonucleic acid (DNA) in particular, as well as extracellular DNA- and RNA-based bacterial TezR receptors or lipopolysaccharides (LPS), have been highlighted as novel and highly specific triggering factors for beta-amyloid and Tau prionogenic aggregation (Tetz et al., 2020; Tetz and Tetz, 2021, 2022a,b; Zhan et al.). The uniqueness of bacterial extracellular DNA and LPS is that they can reach the central nervous system (CNS) either through systemic circulation if the blood-brain barrier is impaired or can be released by microorganisms directly located inside the brain (bacterial presence within CNS is a benchmark of neurodegenerative diseases) (Zhan et al., 2016; Bennett et al., 2019; Dominy et al., 2019; Senejani et al., 2022). For example, high specificity in the DNA of a particular bacterial strain triggered the misfolding of proteins, while DNA of other strains did not. Such specificity could explain the recent failure of a pivotal trial by Cortexyme Inc.; this trial was the first to use brain-localized bacteria as a therapeutic target to treat Alzheimer's disease but failed, possibly due to the overseeing the role of bacterial extracellular DNA as true triggering factor of protein misfolding in this condition (Imbimbo and Watling, 2021).

Another part of the microbiome-based research in neurodegenerative diseases is dedicated to studying the link between the gut microbiota and CNS through the modulation of the enteric nervous system. A review article published in this Research Topic by Geng et al., Shen et al., and Trejo-Castro et al. provided a comprehensive overview of the gut-brain axis in Alzheimer's and Parkinson's diseases, covering the landmark papers of the past decade. Another review in this Research Topic by Li et al. summarized the role of age-related changes in human gut microbiota and neurodegenerative diseases.

Since microbiota plays a critical role in gut-brain axis, a few papers in this Research Topic highlighted the use of different microbiota-targeting products to treat neurodegenerative diseases through the regulation of gut microbiota. Among them, Chung et al. provided an overview of the role of resveratrol in neurodegenerative diseases through the gut-brain axis.

Within the current Research Topic several research articles studying the role of microbiota and neurodegenerative diseases were published. The article published by Aimee Parker et al. reported that normal microbiota prevents dissemination of fungi to the brain in aging animals. Using germ-free mice, the authors have shown that without normal microbiome fungal gut commensals, Candida albicans, an opportunistic pathogen in humans, can traverse the gastrointestinal barrier and disseminate to brain tissue.

Liu et al. published a research article, showing how the interplay of gut microbiota and autophagy participate in the pathogenesis of Parkinson's disease. Another interesting article showed that the microbiota from subjects with neurodegenerative diseases when transferred to animals without these diseases affected the animal brains. Therefore, the transplantation of fecal microbiota from APP/PS1 mice and patients with Alzheimer's to non-affected animals enhanced endoplasmic reticulum stress in the cerebral cortex of wild-type mice (Wang et al.).

Overall, the articles published in this current issue cover the critical topic for the role of microbiota in neurodegenerative diseases. Together with previous data, they pave the way for using brain-localized bacteria and fungi, as well as those located in the gut, as novel potential therapeutic targets for the treatment of these devastating disorders.

## Author contributions

GT analyzed and wrote the manuscript.

## Conflict of interest

The author declares that the research was conducted in the absence of any commercial or financial relationships that could be construed as a potential conflict of interest.

## Publisher's note

All claims expressed in this article are solely those of the authors and do not necessarily represent those of their affiliated organizations, or those of the publisher, the editors and the reviewers. Any product that may be evaluated in this article, or claim that may be made by its manufacturer, is not guaranteed or endorsed by the publisher.
